# Strategies to Block HIV Transcription: Focus on Small Molecule Tat Inhibitors

**DOI:** 10.3390/biology1030668

**Published:** 2012-11-19

**Authors:** Guillaume Mousseau, Susana Valente

**Affiliations:** Department of Infectious Diseases, The Scripps Research Institute, Scripps Florida, 130 Scripps Way, Jupiter, FL 33458, USA; Email: mousseau@scripps.edu

**Keywords:** Tat, TAR, inhibitors, small molecule compounds, transcription, latency, chronic cells, HIV-1, HIV-2

## Abstract

After entry into the target cell, the human immunodeficiency virus type I (HIV) integrates into the host genome and becomes a proviral eukaryotic transcriptional unit. Transcriptional regulation of provirus gene expression is critical for HIV replication. Basal transcription from the integrated HIV promoter is very low in the absence of the HIV transactivator of transcription (Tat) protein and is solely dependent on cellular transcription factors. The 5' terminal region (+1 to +59) of all HIV mRNAs forms an identical stem-bulge-loop structure called the Transactivation Responsive (TAR) element. Once Tat is made, it binds to TAR and drastically activates transcription from the HIV LTR promoter. Mutations in either the Tat protein or TAR sequence usually affect HIV replication, indicating a strong requirement for their conservation. The necessity of the Tat-mediated transactivation cascade for robust HIV replication renders Tat one of the most desirable targets for transcriptional therapy against HIV replication. Screening based on inhibition of the Tat-TAR interaction has identified a number of potential compounds, but none of them are currently used as therapeutics, partly because these agents are not easily delivered for an efficient therapy, emphasizing the need for small molecule compounds. Here we will give an overview of the different strategies used to inhibit HIV transcription and review the current repertoire of small molecular weight compounds that target HIV transcription.

## 1. Introduction

Human immunodeficiency virus type I (HIV-1) is the causative agent of Acquired Immunodeficiency Syndrome (AIDS) and affects more than 30 million people worldwide. Antiretroviral therapy (ART) used to treat the virus is based on triple or quadruple combinations of antiretrovirals (ARVs); although effective and life prolonging, it does not eradicate HIV infection. An HIV RNA level of less than 50 copies/mL of plasma is frequently achieved with ART; however, residual low-level viremia has been detected using ultrasensitive assays [1,2,3,4]. HIV persists in stable reservoirs harboring an integrated form of the HIV genome, wherein continuous viral production and reactivation of transcription from these reservoirs are not affected by current ARVs [5,6]. As such, novel classes of ARVs are needed to inhibit these processes. 

**Figure 1:**
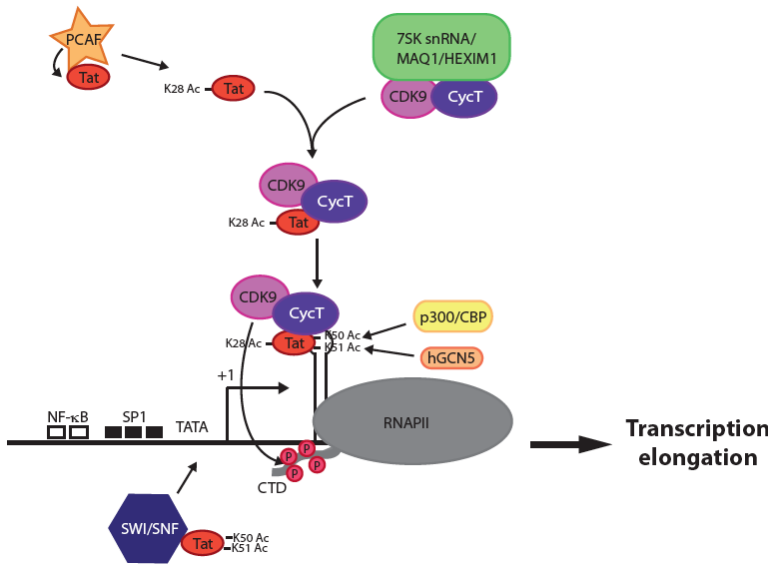
Upon Tat acetylation on Lys28 by PCAF, Tat recruits P-TEFb (CDK9/Cyclin T1) from its inactive complex with 7SK snRNA/MAQ1/HEXIM1. Tat/P-TEFb complex binds to TAR. CDK9 phosphorylates Ser2 of the RNAPII CTD stalled shortly after transcription initiation and promotes transcription elongation. Tat is acetylated at Lys50 and Lys51 by p300/CBP and hGCN5, resulting in the release of the complex from TAR. Tat then recruits to the initiation start site SWI/SNF, PCAF and RNAPII and other factors not depicted here to promote further transcription.

An important step in the replication of HIV-1 is the reverse transcription of the viral genomic RNA into cDNA and the integration of the proviral genome into the host chromosome. Efficient HIV-1 gene expression is dependent upon the viral protein Tat during the exponential growth of the virus and for robust transcription of the integrated proviral genome. Tat is a 14 kDa, 101-amino acid protein that is initially expressed from rare full-length transcripts that are multiply spliced. In the absence of Tat, transcription initiates normally at the 5' long terminal repeat (LTR) promoter, but results in short, abortive viral transcripts due to RNA polymerase II (RNAPII) pausing shortly after promoter clearance [7]. Tat controls transcription at the level of RNAPII elongation through interaction with the TAR RNA (an RNA stem loop structure that spontaneously forms within the first 59 nucleotides of each viral transcript) [8,9] and the positive transcription elongation factor b (P-TEFb), composed of Cyclin T1 and Cyclin-dependent kinase 9 (CDK9) (Figure 1) [10,11]. In the absence of Tat, P-TEFb exists in the cell as a large inactive complex composed of 7SK snRNA and MAQ1/HEXIM1 proteins [12,13]. The recruitment of P-TEFb by Tat leads to several phosphorylation events carried out by CDK9 that convert paused elongation complex to a highly processive form. CDK9 phosphorylates Ser2 of the RNAPII C-terminal domain (CTD) heptapeptide repeat, allowing interaction with additional factors involved in productive transcription (reviewed in [14]). The result of these post-translational modifications is synthesis of high levels of full-length viral transcripts.

Proviral promoter activity is directly governed by its chromatin environment [15]. Histone post-translational modifications, including phosphorylation, acetylation, methylation, and ubiquitination, contribute to transcriptional regulation by defining an “open” or “closed” state of chromatin [16]. Nuc-1, a nucleosome located immediately downstream from the transcription start site, directly impedes LTR activity [17,18]. Additional blocks at the level of transcription initiation are imposed by specific epigenetic chromatin modifications at nucleosomes on the 5' LTR, notably, deacetylation and methylation of histone *N*-terminal tails. Hallmarks of HIV-1 latency are low levels of Tat [19] or P-TEFb [20] and a sustained production of prematurely terminated RNA transcripts [21,22]. Entry into latency also requires recruitment of Histone Deacetylase 1 HDAC1 [23,24], histone methyltransferase Suv39H1 and heterochromatin protein HP1 [25] to the chromatin surrounding the HIV-1 LTR. Transcriptional reactivation is accompanied by changes in the local chromatin structure, which is accomplished by recruitment via Tat of chromatin remodeling factors such as SWI/SNF [26,27] and histone acetyl transferases (HATs), such as CREB binding protein CBP and p300 [28], p300/CBP associated factors (PCAF) and the histone acetyl transferase hGCN5 [27,29,30,31,32,33], where they can reverse the effects of histone deacetylation (Figure 1). Lys28 and Lys50 of Tat are targets of acetylation by PCAF and p300, respectively [30,33]. Acetylation of Lys28 enhances Tat binding to P-TEFb, whereas acetylation of Lys50 dissociates Tat/P-TEFb from TAR, indicating a two-step, time-dependent mechanism for transcriptional regulation by Tat associated histone acetyltransferases (Figure 1).

In this review, we will focus on small molecule inhibitors that inhibit Tat-dependent transcription. We will also briefly discuss the different strategies used to inhibit HIV transcription by targeting other transcription factors (CDK9, Cyclin T1, NF-kB, Sp1), as well as non-small molecule inhibitors (Tat vaccines, RNAi, ribozymes, peptides-based or oligonucleotide based inhibitors). We will discuss the screening strategies that were used to identify small molecule inhibitors targeting Tat or Tat/TAR interaction and the compounds obtained from these efforts. Finally, we will give a brief overview of a novel and recently identified analog of the natural compound Cortistatin A isolated from a marine sponge, that inhibits Tat-dependent transcription in acutely and chronically HIV infected cells at sub-nanomolar concentrations without cell associated toxicity.

## 2. What Can We Expect from a Tat Inhibitor and What Features Make Such a Compound Different from Current ARVs?

Since Tat does not have any apparent cellular homolog, it is a very attractive target to block specific HIV transcription, and as Tat is expressed early on in the replication cycle, its inhibition would also control transcription of other viral genes expressed later. 

Transcription of the HIV-1 provirus is regulated by both viral and cellular transcription factors. Before Tat is produced, low-level basal transcription from the viral promoter is initiated by cellular factors, such as NF-kB [34], Sp1 [35], TATA-binding protein [36] and RNAPII. A desirable Tat inhibitor should block Tat-mediated activation of the viral promoter without affecting its basal transcription, given the shared transcription factors of the HIV promoter and cellular genes. Therefore, a maximal expected reduction of viral replication by 80%–90% in acutely infected cells should be expected, as cellular activation during acute infection triggers promoter activation independently of Tat, and should not be compared with the usual multi-log reduction observed with protease inhibitors. However, in prolonged treatment of chronically infected cells, a total shut-off of residual transcription can be achieved. This reflects a unique feature of a Tat inhibitor, as it blocks transcription of the integrated provirus, contrary to the other anti-HIV drugs that only block *de novo* infection. The reduction of residual viral replication from chronically or latently infected cells may establish a perpetual latent state. Such transcriptional shut-off may reduce the pool of the latently infected cells by diminishing reservoir replenishment, which may accelerate the eradication of the latent reservoir. Furthermore, one would expect that in the presence of a Tat inhibitor, it would be very difficult for external stimuli such as antibodies (αCD3/CD28), or phorbol esters (PMA) or HDAC inhibitors to reactivate virus production from the integrated provirus, as it is known that HIV-1 lacking Tat undergoes some basal transcription; however, it does not sustain a spreading infection [37].

HIV-1 is categorized in three main groups: M (major), O (outlier) and N (non-M/non-O), where group M is additionally subdivided into subtypes (or clades) A–D, F–H, J and K [38,39]. Variations between subclades of viruses may play an important role in their pathogenesis. Nucleotide variations within the LTR promoter region of main subtypes B, C and E include alterations in the TATA box, the NF-kB enhancer, the TAR element, as well as Sp1 binding sites [40,41]. The HIV Tat protein also exhibits amino acid divergence among the different clades, which may influence binding and transactivation functions. An ideal Tat inhibitor would be able to inhibit Tat activity irrespective of its clade.

Finally, Tat can also be released from HIV-infected cells and alter several functions in uninfected cells. In the brain, Tat induces neuronal dysfunction/toxicity, even though neurons cannot be directly infected with HIV, resulting in central nervous system (CNS) pathology (reviewed in [42,43]). An ideal Tat inhibitor would also impact these other Tat mediated activities.

## 3. Targets and Strategies: Approaches to Reduce HIV Transcription

### 3.1. Targeting Cellular Factors Involved in HIV Transcription

Several cellular components may serve as potential targets for antiviral chemotherapy [44]. The complex P-TEFb is one of them, but finding a highly selective and non-cytotoxic CDK9 inhibitor is a difficult task due to its role in cellular transcription [45,46]. Nevertheless, CDK9 has been the focus of several studies (Reviewed in [46,47,48]). Among the principal inhibitors known to block CDK9 kinase activity is flavopiridol [49,50] and derivatives [51], indirubin-3'-monoxime [52,53], a nucleotide analog DRB [54], and R-roscovitine (CYC202) [54]; however, this last one is also known to inhibit other CDKs, such as CDK2 [55]. Recently, iron chelators have also been shown to block HIV-1 transcription by targeting both CDK9 and CDK2 [56].

Cyclin T1 has also been the target of several inhibitors such as anti-human Cyclin T1 intrabodies targeting the Cyclin T1/Tat interaction [57], microRNAs targeting Cyclin T1 expression [58], dominant negative mutants of Cyclin T1 that act by either specifically degrading Tat [59] or forming kinase inactive complexes with Tat and CDK9 [60]. The growth factor granulin and some of its granulin cysteine-rich motif repeats were able to inhibit Tat transactivation by either binding Tat or the histidine rich domain of Cyclin T1 [61,62]. Potent inhibition of Tat transactivation is obtained by the overexpression of HEXIM1 (which sequesters P-TEFb in an inactive form) or its paralog HEXIM2 by binding to Cyclin T1 [63]. Furthermore, the human I-mfa domain-containing protein (HIC), as well as its I-mfa domain alone, can act as a dominant negative repressor [64]. 

CDK2 is another possible target to block HIV transcription. Indeed, Tat interacts with both the CTD of RNAPII and CDK2/Cyclin E, and hence helps CTD phosphorylation at Ser2 by CDK2. This kinase was suggested to be required for Tat-dependent transcription *in vitro* [65,66]. Among CDK2 inhibitors that blocked HIV-1 transcription were CYC202 ([55]; reviewed in [67]), Alsterpaullone [68], a CDK2 RNAi [69], and small peptide inhibitors [70,71]. CDK2/Cyclin E was also suspected to be implicated in the HIV transcription inhibition by an *N*-aminoimidazole derivative NR-818 [72].

Transcription factors such as Sp1 and NF-kB play a critical role in initiation of HIV transcription in newly infected cells when Tat has not yet been made [73]. They are potential targets to block HIV basal transcription [74]; however, off target effects might be observed due to the shared nature of these transcription factors with cellular genes. A plant lignan, isolated from *Larrea tridentata* [75] and derivatives [76,77], inhibited basal transcription by preventing interaction of Sp1 to its DNA binding site as well as Tat-dependent HIV replication in peripheral blood mononuclear cells (PBMCs). Some compounds have also been reported to interfere with both Sp1 and NF-kB [78,79,80]. Several studies have identified and characterized compounds targeting NF-kB-mediated activation of HIV-1 transcription, but none of them have reached the necessary selectivity (Reviewed in [74,81]). More recently, two inhibitors of tumor necrosis factor α (TNF-α) production, piperidylpyrimidine [82] and LMP-420 [83], have been shown to inhibit respectively HIV-1 LTR transactivation or/and HIV replication in PBMCs. Another known inhibitor of TNF-a production and HIV replication, pentoxifylline, initially found to inhibit HIV by blocking the binding of NF-kB to its motif [84,85], was shown to block HIV by inhibition of a cellular DNA damage sensing and repair protein, ATR [86,87]. Finally, noraristeromycin, a carbocyclic adenine nucleoside, inhibits IkBa phosphorylation and degradation, thus preventing phosphorylation of p65, one of the NF-kB subunits [88]. 

P300/CBP is also a potential target due to its role as co-activator of Tat transcription, as well as in transcription reactivation by acetylating Tat and by reversing effects of histone deacetylation [30,33]. A specific inhibitor of p300/CBP HAT activity, such as curcumin, blocked acetylation of Tat *in vitro* and the proliferation of the virus by reducing HIV transcription [89]. Another inhibitor, LTK-14, was shown to be more specific and less toxic than its parent garcinol by inhibiting only p300 and not PCAF, but did display some toxicity by altering global gene expression [90,91]. LTK-14 blocked histone acetylation in HIV infected cells and restricted HIV at micromolar concentrations. Another potential cellular target is PCAF, a Tat recruited HAT, which is involved in reactivation from latency. A polyclonal PCAF BRD antibody blocked Tat transactivation [92]. A few thousand compounds have been tested on an NMR-based chemical screen yielding one lead compound, and upon structure activity relationship (SAR), N1-aryl-propane-1,3-diamine was found to selectively bind to the BRD domain of PCAF [93]. Using a structure-based lead optimization of small molecule, Pan *et al*. found a compound able to disrupt Tat/PCAF interaction and Tat-dependent transactivation in the micromolar range [94].

Tat activates NF-kB via oxidative stress-induced cell signaling pathways like the PI3K/Akt signaling pathway [95]. Epigallocatechin-3-gallate by activating both AMPK and Nrf2 antioxidant signaling pathways, leads to the inhibition of the AKT signaling pathway preventing both NF-κB activation and Tat induced HIV-1 transactivation [96]. Another compound targeting the Akt pathway is the coumarin derivative BPRHIV001, displaying a therapeutic index (TI) (half-maximal cytotoxic concentration (CC_50_)/half-maximal inhibitory concentration (IC_50_)) over 1,000 in acutely infected PBMCs [97]. This compound decreases the phosphorylation of PDPK1, a known Akt activator, which in turn blocks Akt phosphorylation, leading to a destabilization of p300 protein, thus negatively influencing Tat-dependent transcription. Alternatively, 9-aminoacridine activates the Akt pathway and phosphorylation of p21WAF1, reactivating p53 and p21WAF1 pathways, leading to HIV replication inhibition. Indeed, p21WAF1 associate with P-TEFb, which leads to a decrease of CDK9 on the HIV LTR and inhibits Tat-dependent transcription [98].

Overexpression of NIPP1, the major nuclear inhibitor of the host Ser/Thr phosphatase-1 (PP1) [99], or stable expression of its central domain [100] increased CDK9 phosphorylation on Thr186 and the association of CDK9 with 7SK RNA, as well as decreased binding of PP1 to Tat. Compound 1H4, found by screening a library of 300,000 compounds, decreases transcription and HIV replication by its ability to bind a RVxF motif on PP1 and to disrupt PP1/Tat interaction at micromolar concentration, which are unfortunately close to being toxic [101]. Finally, resveratrol, a polyphenolic phytostilbene found in grapes and berries, was able to decrease Tat-induced HIV-1 LTR transactivation in a TAR-independent manner by increasing NAD^+^ and SIRT1 production [102,103,104]. Indeed, SIRT1, a class III HDAC, is known to recycle Tat into an inactive form by deacetylating Lys50 [105]. Tat is also known to inhibit SIRT1 activity, that in turn blocks the deacetylation of the NF-κB p65 subunit, resulting in transcription hyperactivity [105,106].

### 3.2. Non-Small Molecules Compounds Used to Inhibit Tat/TAR Mediated HIV Transcription

Oligonucleotide derivatives such as TAR decoys, aptamers, ribozymes and RNA interference (RNAi) have been the focus of numerous studies to inhibit Tat-dependent HIV transcription (reviewed in [107,108]). Recently, several clinical trials have been done targeting total or partial HIV transcription using TAR decoys, aptamers, ribozymes and/or small interference RNAs (siRNAs) [109]. The hammerhead ribozyme (OZ1) targeting the overlapping HIV vpr and Tat coding sequence has been delivered into primary cells using a gammaretroviral vector, an approach that is currently in phase II clinical trials ([110,111]; reviewed in [112,113]). RNAs have been designed to target conserved viral sequences in order to silence gene expression at the post-transcriptional level [114,115]. One of the major difficulties is delivery of the siRNAs into the appropriate target cells. To circumvent this issue, chimeric RNAs containing a CD4 aptamer and an siRNA have been designed allowing for the specific delivery into CD4 expressing cells, and via receptor competition, inhibit HIV entry, as well as gene expression. Others, such as anti-gp120 aptamer-siRNA chimeras, also display this inhibitory behavior (reviewed in [115]). An alternative delivery approach is to use RNAi-based gene therapy using lentiviral vectors to transduce RNA based inhibitors into non-dividing cells and thus, permanently deliver the transgene. To avoid the selection of resistant mutants, several RNA-based inhibitors can be used together. RNA-based hematopoietic cell gene therapies for treatment of HIV infection using ribozymes, aptamers and siRNA have been reviewed [116]. A lentiviral vector encoding a combination of a TAR-decoy (small nucleolar RNA that binds the Tat protein), a CCR5 targeting hammerhead ribozyme and an shRNA targeting Tat-Rev mRNAs suppressed HIV replication for over 42 days in primary CD34^+^ hematopoietic stem cells ([117]; reviewed in [114]). Preliminary proofs of safety using RNA-based hematopoietic cell gene therapy have been obtained; a study showed that transgenic T cells generated in SCID-hu-mouse have normal T-cell phenotypes [118]. This lentiviral vector has been used in phase I clinical trials, via *ex vivo* gene therapy, using hematopoietic stem cell engraftment in human patients [119]. Foamy virus vectors, another type of virus based vector, is also being explored. A foamy virus combinatorial anti-HIV vector that expresses three anti-HIV transgenes (HIV fusion inhibitor and two shRNAs against either Tat or Rev) was successfully transduced into CD34^+^ cells and engrafted into SCID mice [120]. Despite lingering challenges in delivery and off-target toxicity, RNA-based hematopoietic cell gene therapies are promising approaches to fight HIV.

Peptide-related inhibitors, such as peptoids [121] and Tat-mimicking peptides, have been developed to block Tat-dependent HIV transcription (reviewed in [74,107]). More recently, L50 a cyclic b-hairpin peptide was found to bind to TAR with nanomolar affinity and induce a conformational change in the bulge region that was incompatible with Tat binding [122,123,124]. However, L50 inhibited HIV replication in PBMCs in the high namolar range and affected reverse transcription and HIV transcription in the high micromolar range. So far, no peptide-based inhibitor has entered clinical trials. Interestingly, T-U2AF65, the fusion of a splicing factor with the Tat activation domain, showed a dominant negative effect on HIV transcription. This inhibitor binds to RNAPII CTD and to Cyclin T1, blocking the interaction of wild type Tat to the CTD in a TAR-independent fashion [125]. Expression of T-U2AF65 slowed down virus replication in a lymphocytary cell line, which eventually became chronic, but replicated poorly.

Besides its important role in viral transactivation, extracellular Tat is an important factor in viral pathogenesis. Strategies to develop a vaccine using Tat as an epitope have been reviewed [107,126,127]. Several clinical trials have been done to assess therapeutic and preventive Tat-based vaccinations [128,129,130,131,132]. TUTI-16, a synthetic anti-Tat epitope, successfully induced high titer antibodies against Tat variants from all clades and was tested on asymptomatic HIV infected subjects in phase I/IIa clinical trials [133,134]. TUTI-16 significantly reduced viral load at low vaccine doses, but not at higher doses due to activation of cytokines by the adjuvant components. In another phase I/IIa clinical trial, HIV-infected patients were vaccinated with mRNA electroporated dendritic cells expressing Tat, Rev and Nef. This vaccine was shown to be safe and feasible, but had no significant impact on clinical parameters [135]. The HIV-1 Oyi strain was identified in HIV infected patients in Africa who did not progress to AIDS and, though genetically similar to common HIV-1 strains, contained mutations in the Tat gene not found in other Tat variants. Using Tat Oyi, Mediouni *et al*. successfully developed a monoclonal antibody named 7G12 that has the capacity to recognize heterologous Tat variants by a common 3D epitope [136]. This antibody was able to block the uptake of extracellular Tat, resulting in the inhibition of all Tat biological activities. Despite several trials, vaccine based therapies have been met with limited success.

## 4. Small Molecules Inhibitors of HIV Transcription

### 4.1. Methods Used to Discover Small Molecule Inhibitors of Tat-Dependent Transcription

#### 4.1.1. Biochemical Methods

Several strategies have been used to find small molecule inhibitors of Tat-dependent HIV transcription. Pharmacological compounds have been tested randomly, based on some known characteristics such as DNA or RNA binding properties [137,138,139,140] or because they are known inhibitors of other viruses [141,142,143]. Others have been tested due to their low toxicity and good tolerance in mice or patients. For example, topotecan was able to inhibit 80% of HIV-1 p24 antigen production in chronically infected cells at low nanomolar range with a dose 200-fold lower than what is well tolerated by cancer patients [144]. Sometimes serendipity plays a role, e.g., a search for inhibitor compounds of a kinase implicated in inhibiting HIV mRNA 3' end processing [145] turned out to specifically inhibit Tat-dependent HIV transcription [146].

Nevertheless, most of the compounds have been screened biochemically, looking at their ability to bind TAR or to disrupt Tat/TAR binding using techniques such as electrophoretic mobility shift assay (EMSA), filter binding assay, scintillation proximity assay (SPA) and ESI-MS (all described in [147,148]). EMSA or filtration assays, followed by an *in vitro* LTR-reporter cell-based assay identified inhibitors of Tat/TAR interaction and Tat-dependent transcription, as is the case for furimidazoline (DB60) [149], CGP 40336A [150], aromatic polyamides TAPB and TAPP (resembling the DAPI structure) [138], antibiotics (neomycin, streptomycin and gentamicin) [151] and a Tat/TAR inhibitor issued from a screen of 150,000 compounds [147].

#### 4.1.2. LTR Reporter Activity

Numerous studies have used stable cell lines expressing the HIV promoter 5' LTR, driving a reporter gene such as luciferase (Luc), b-galactosidase (LacZ), green fluorescent protein (GFP), alkaline phosphatase (SEAP) or chloramphenicol (CAT) co-transfected with a plasmid expressing Tat to either validate inhibitors of Tat/TAR binding or to screen for new compounds for inhibition of HIV transcription. The first compound described to inhibit Tat-dependent transcription was a benzodiazepine derivative, Ro 5-3335, found during a random library screen using an LTR-gene reporter assay [152]. The potency of Ro 5-3335 and its less toxic derivative Ro 24-7429 [153] was highly dependent on the nature of the assay and the cell type used. Indeed, these compounds were effective in the micromolar range in acutely infected PBMCs and macrophages, but were less effective at inhibiting HIV replication in chronically infected macrophages, a major viral reservoir. Besides, these compounds had no effect on acutely infected MT-2 and MT-4 cells or persistently HIV-1 infected cells (HUT78/IIIB/LAI) [154,155]. Ro 24-7429 was found most likely to inhibit the activity of a TAR binding cellular factor [153,156]. Clinical trials were initiated; however, due to nervous system side effects and absence of activity in patients, these efforts were cut short. Though benzodiazepine derivatives were not successful in terms of selectivity for Tat and activity in patients, the screening of small molecule inhibitors against Tat gave the first example that they can inhibit transcription in some types of acutely, chronically and latently HIV infected cell lines. Therefore, several other studies have used LTR-reporter assays to find Tat-dependent transcription inhibitors such as keto/enol epoxy steroids [157], purine derivatives [158,159], tanic acids [160], D-penicillamin [161,162], organic thiophosphate WR-151327 [163], b-carbolines [139,140], camptothecin [144,164], durhamycin A [165], monocyclin IV [166], 3-phenylcoumarins and chalcones [167], stilbene related heterocyclic compounds [168], 4-phenylcoumarins and derivatives (both anti NF-kB and Tat activities) [169], topotecan, b-lapachome, [144], toco-pheryl curcumin (C3) [170], the cyclopentanone prostaglandin PGJ_2_ [171,172], celasterol [173], bis-anthracycline antibiotics (WP 631; a DNA intercalator) [137], the natural product EM2487 [174], thiamine disulfide [175] and 2-glycineamide-5-chlorophenyl-2-pyril ketone [176]. Recently, Chande *et al*. have constructed a plasmid and a lentiviral vector harboring both an LTR-driven reporter cassette and a Tat gene under the control of a viral promoter to establish stable cell lines [177]. This updated method provided an easy one-step way to test inhibitors of Tat-dependent transcription. However, it is important to keep in mind that LTR-reporter gene assays may also lead to identification of off-target inhibitors affecting other cellular factors important to HIV transcription (for examples [153,178]).

#### 4.1.3. Alternative Methods

To develop TAR-binding antiviral drugs, some groups have performed ligand-based screening by NMR using TAR as target, locked by a specific chemical probe into a conformation favorable to compound binding [179]. Others have used fluorescence resonance energy transfer (FRET) to look at inhibitors of Tat/TAR binding [180]. Hwang et al. used a bead colorimetric screening assay for TAR RNA binding ligands that enabled the discovery of compounds inhibiting HIV replication in the micromolar range [181]. A fluorescent high-throughput assay was aimed to select TAR binding molecules that compete a described TAR-binding peptoid/Europium-labeled TAR interaction. Screening of 200,000 compounds has identified a highly specific stilbene derivative CGA137053 that disrupts Tat/TAR binding in the nanomolar range and inhibits Tat transactivation, as well as HIV infection in macrophages and PBLs [182,183]. The interaction between TAR and a fluorescently labeled Tat peptide was used to test neamine derivatives [184,185]. Finally, to develop new transcription inhibitors, several studies have used structure activity relationship (SAR) to further optimize their best lead (for example [186,187]).

#### 4.1.4. Docking Study

As an alternative to standard high-throughput screening, several groups have used docking strategies to design and/or screen *in silico* new compounds against Tat/TAR interaction. Lind *et al*. performed a computational virtual screening based on a TAR 3D structure of approximately 181,000 non-peptide/non-nucleoside compounds from the Available Chemicals Directory (ACD) and found that acetylpromazine disrupts Tat/TAR binding in the nanomolar range [188], an activity latter reported to be only effective in the high micromolar range [189,190]. Interestingly, *in silico* screening of the ACD yielded a high number of all known TAR ligands of the library (aminoglycoside antibiotics, L-Arg and spermine), as well as new potential compounds [191]. Alternatively, a virtual screening has been performed to compare potential ligand properties against a known potent Tat-TAR inhibitor used as reference. The best hits were validated on Tat/TAR interaction by FRET [190]. Schuller *et al*. designed compounds by assembling acetylpromazine as a template structure with unique chemical blocks to generate novel ligands of TAR RNA [192]. However, all the compounds from these studies inhibited, at best, the Tat/TAR interaction in the micromolar range and have not been tested on replicating HIV. More recently, to find new TAR binding small molecules, Stelzer *et al*. have developed software using molecular dynamic to dock compounds onto a RNA dynamic ensemble, including twenty TAR conformers. This helped solve the problem of taking into account large degrees of RNA conformational adaptation during virtual screening [193]. Out of 51,000 *in silico* structures tested, several hits were confirmed to bind TAR and inhibit its binding to Tat by fluorescence based binding assay. The highest specificity to bind TAR was achieved by netilmicin, an aminoglycoside antibiotic. This compound is able to block acute HIV-1 replication by 81% in T-cell lines and HIV replication in an HIV-1 indicator cell line with an IC_50_ comparable to other known Tat/TAR inhibitors. However, aminoglycoside derivatives were never shown to have any specific mechanism of action against HIV, and this might also be the case for netilmicin [194]. Nevertheless, by finding the first experimentally validated RNA-targeting compound, these studies demonstrate that *in silico* screening might be a good alternative to standard high-throughput screening.

### 4.2. A Selection of Transcription Inhibitors with TI > 100

Natural aminoglycoside antibiotics such as neomycin have been shown to interact with TAR and to inhibit HIV, but non-specifically [151]. To increase affinity and selectivity to TAR, derivatives have been made by adding to the aminoglycoside backbones Arg or guanidino groups [184,195,196,197]. The best compound of the family is NeoR, an aminoglycoside derivative with a TI of 125 in PBMCs (Table 1) [197]. However, L-Arg-aminoglycoside conjugates were not completely selective in inhibiting HIV transcription, since they also blocked the virus entry into cells by interacting with the CXCR4 coreceptor [198,199]. Mechanisms of inhibition by amino-glycoside derivatives of virus entry or HIV transcription, as well as comparison to the compounds available, have been recently reviewed [194]. Moreover, aminoglycoside-Arg conjugates seemed to also be general inhibitors of eukaryote and prokaryote translation [200].

Temacrazine, a nanomolar active bis-triazoloacridone compound with low toxicity, was initially tested because of its anti-transcriptional activity in cancer and was found to inhibit HIV in acute, chronic and latent cells (Table 1) [201]. It is a selective inhibitor of transcription and blocked several strains of HIV and RT resistant mutants; however, it did not affect HIV-2 or SIV. Moreover, the mechanism is unknown, since it did not seem to affect Tat/TAR, Rev/RRE interactions and TNF-a pathway. It was suggested it affects a still unidentified highly specific viral target required for HIV-1 transcription. Besides this publication, no further work on HIV inhibition by temacrazine was ever reported, suggesting that this compound might present some undesirable properties to be pursued as an anti-HIV drug.

Several reports show that quinolone derivatives presented anti-HIV properties (reviewed in [202]). One of the early reports identified fluoroquinoline, K12, as a selective inhibitor of HIV transcription in acute, chronic and latent cells [203,204]. However, its mechanism of action was TAR independent and did not seem to be mediated by TNF-a, Tat or Sp1 [205]. WM5, a 6-aminoquinolone, was able to inhibit Tat-dependent LTR-driven transcription at the micromolar range, as well as specifically blocking Tat/TAR interaction by binding to TAR (Table 1) [187,206,207]. Using WM5, a 6-aminoquinolone, as a lead compound, several derivatives have been synthesized during several successive SAR (reviewed in [202]). The best quinolone based inhibitors of Tat-dependent transcription described so far are HM13N and NM13 (Table 1) [186,208]. HM13N, a drug-like compound, was obtained by replacing the quinolone ring with a naphthyridone core. This naphthyridone derivative was able to inhibit both HIV-1 and HIV-2 in MT-4 cells and displayed a reasonably good TI in chronically and latently infected cell lines, but was less potent in acutely infected Jurkat cells and PBMCs. The second compound, NM13, was selective only to HIV-1 with an IC_50_ of 80 nM and a therapeutic index ³3,707 in MT-4 cells [208]. However, this compound presents a low solubility as compared to HM13N. It is interesting to note that two related 6-desfluoroquinolones, HM12 and HM13, were also able to inhibit *in vivo* TNF-a reactivation of latently infected OM-10.1 cells engrafted in hu-SCID mice [209,210]. However, to our knowledge, after more than 10 years of quinolone derivative optimizations, none of these compounds ever reached clinical trials, due in part to either solubility or cytoxicity issues.

Tat inhibitors were also screened from natural products of microbial fermentation extracts [165]. Durhamycin A, from the aureolic acid antibiotics family, is closely related structurally to the anti-tumor antibiotic UCH9. When tested on Jurkat cells infected with a defective HIV-1 virus needing co-transfection of Tat, the viral replication was inhibited in the low nanomolar range with a TI > 5,000 (Table 1). However, this molecule, with a MW over 2,000, is quite large, limiting its utility as an anti-HIV drug.

### 4.3. Didehydro-Cortistatin A (dCA)

**Figure 2:**
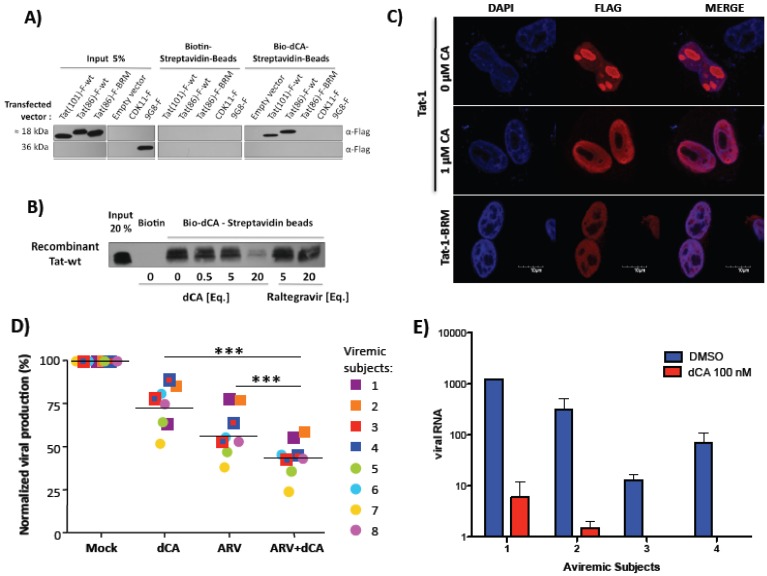
dCA inhibits Tat-mediated trans-activation of the integrated provirus by binding specifically to the TAR-binding domain of Tat. (**A**) A biotinylated form of the compound (Bio-dCA) coupled to streptavidin-coated magnetic beads retained Tat-wt, but not TAR non-binding mutant of Tat. HEK293T cells were transfected with flag-tagged Tat 101 aa (Tat-F-101-wt), a shorter Tat version with 86 aa (Ta(86)-F-wt), Tat 86 aa mutated in the basic domain (Tat(86)-F-BRM), flag-tagged CDK11 (CDK11-F), flag-tagged 9G8 (9G8-F) and empty vector control. Protein extracts were incubated with Streptavidin beads coated with either Biotin or Bio-dCA. Pulled-down proteins were revealed by western bloting. (**B**) Pull-down experiments with recombinant Tat purified protein bound directly to Bio-dCA and was competed by dCA, but not by Raltegravir, used as negative control, demonstrating the specificity of the interaction. (**C**) Confocal microscopy analysis of the subcellular localization of transfected Tat(86)-F-wt and Tat(86)-F-BRM, treated or not with dCA. Magnification, 300×. (**D**) dCA activity on CD4^+^ T cells from viremic and aviremic HIV-infected subjects. The potency of dCA was tested on spontaneous viral output (p24 production analyzed by ELISA) from primary CD4^+^ T cells isolated from 8 viremic HIV-infected subjects. The inhibition of viral replication mediated by dCA alone [100 nM (circle) or 1 mM (square)] was approximately 25%. ARVs (AZT (zidovudine), EFV (Efavirenz) and RAL (Raltegravir)), which inhibit all new infections, decreased viral production by approximately 40%, while dCA and ARVs combined inhibited approximately 60%. (**E**) CD4^+^ T cells isolated from four subjects who had been treated with ARVs for at least three years who spontaneously released viral particles *in vitro* were cultured for six days in the presence or absence of dCA and ARVs to block *de novo* infection. Using an ultrasensitive RT-qPCR assay, in the presence of dCA, we observed a reduction of viral production of 99.7% at day 6. Reprinted from [146], with permission from Elsevier.

We have recently found that dCA, an analogue of a natural steroidal alkaloid from a marine sponge, inhibits Tat-mediated trans-activation of the integrated provirus by binding specifically to the TAR-binding domain of Tat (Figure 2A,B) (Table 1) [146]. dCA represses transcriptional activation when transfected Tat or purified recombinant Tat is added to cells stably expressing the 5' LTR promoter driving Luciferase expression. When transfected, Tat localizes in the nucleus and specifically accumulates in the nucleolus. dCA causes redistribution of Tat to the periphery of the nucleolus, forming a distinctive ring-like structure determined by confocal fluorescence microscopy of HeLa-CD4 cells expressing HIV-1 Tat-flag (Figure 2C). Without a TAR binding domain, the basic region-mutant Tat-1-BRM is excluded from the nucleolus, again confirming that dCA binds Tat via its TAR binding domain. By chromatin immunoprecipitation (ChIP), we demonstrate that dCA inhibits transcription initiation and elongation from the viral promoter in chronically infected cells [146]. 

**Table 1:** Activity, cytotoxicity and therapeutic index of selected HIV-1 Tat-dependent transcription inhibitors. Acute, chronic and/or latent cell lines where HIV-1 has been significantly inhibited are listed. The type of screen, when applicable, and the compound structure are indicated. (* Note: The IC_50_ and CC_50_ have not been determined in every cell lines listed). N.A.: not applicable.

Compound	Structure	IC_50_ (nM)	CC_50_ (mM)	TI	Tested in	Cell types*	Screen	References
dCA	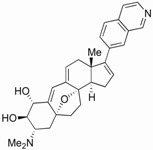	0.0007-2.6	20	>8000	Acute, Chronic	Hela CD4+, T cell line, PBMCs, CD4+ T cells	N.A.	[146]
Durhamycin A	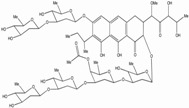	4.8-11	5-25	431-5208	Acute	Hela CD4+, T cell lines	LTR-reporter assay	[165]
WM5	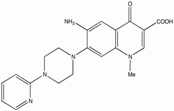	30-850	2.21 to > 263	15->3333	Acute, Chronic	Hela, T-cell lines, PBMCs	SAR	[187,206,207,211]
NM13	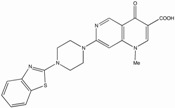	80	³296	³3707	Acute	T-cell line	SAR	[91,208]
HM13N	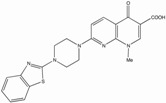	4-1200	1.5-26.33	17-1333	Acute, Chronic, Latent	T-cell Lines, monocytic cell lines, PBMCs	SAR	[186]
Temacrazine	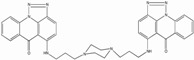	0.1-72	1-10	21-2518	Acute, Chronic, Latent	T cell line, monocytic cell lines	N.A.	[201]
NeoR	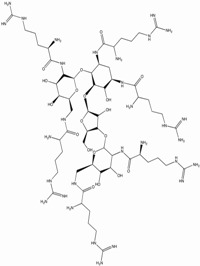	1.7-5.3	275-500	33-250	Acute, Chronic	T-cell line, promonocytic cell line, PBMCs	SAR	[197]

Working at subnanomolar concentrations, dCA inhibits wild type HIV-1 and HIV-2 replication in acutely and chronically infected cells at an IC_50_ as low as 0.7 pM. By comparison, selected compounds reported in Table 1 inhibit HIV only in the low-to-high nanomolar range (Table 1). Consistent with this mode of action, dCA treatment for 10 or 60 days of chronically infected cells reduces viral RNAs to undetectable levels with an IC_50_ of »0.1 nM and IC_90_ of <10 nM [146]. Moreover, dCA provides a significant additive effect with other ARVs when added to primary CD4^+^ T cells from HIV-viremic subjects (Figure 2D). Importantly, dCA abrogates spontaneous residual viral particle release from CD4^+^ T cells from virally suppressed subjects on highly active antiretroviral therapy (HAART) (Figure 2E). Finally, dCA discontinuation does not result in viral rebound [146]. Indeed, after terminating dCA treatment of HeLa-CD4 cells chronically infected with pNL43, no virus rebound was observed even 27 days post treatment arrest, contrary to what is observed with ARVs, suggesting that dCA promotes rapid and prolonged silencing of the promoter. Thus, dCA defines a unique class of anti-HIV drugs that may inhibit viral production from stable reservoirs and reduce residual viremia during HAART.

## 5. Conclusions

Many methods have been employed to screen for Tat-dependent transcription inhibitors. Screening for Tat/TAR inhibitors using, for example, EMSA or filtration assays allowed the discovery of compounds with a good constant of inhibition and good affinity for TAR, however if tested against HIV replicating virus, most of the time they displayed activity only in the micromolar range with low TI. LTR-reporter assay with co-expression of Tat is a widely used tool to screen for transcription inhibitors. However, this type of assay may identify non-Tat specific inhibitors that affect cellular, factors often resulting in toxicity. So far, besides Ro 24-7429, no small molecule inhibitors of Tat or TAR have entered clinical trials.

From the findings described herein, dCA is the most potent anti-Tat inhibitor described to date. It binds selectively to the basic domain of HIV Tat, a region also responsible for the Tat-TAR interaction. Importantly, dCA has a drug-like structure, is highly soluble in water and displays good bioavailability in mice. dCA inhibits both HIV-1 and HIV-2 replication in tissue culture-adapted cells or in primary cells when used at single digit nanomolar concentrations with no associated toxicity at the cellular level. The dose-response curve for dCA is unusual and flattens out, resulting in partial inhibition in acute infections. However, even though dCA alone fails to totally inhibit acute HIV infections, due to residual Tat-independent promoter activity, this feature is desirable as it limits off-target effects from shared transcription factors binding cellular and viral promoters, such as NF-κB.

dCA treatment was extremely successful at reducing viral production by a drastic 99.7% from primary CD4^+^ T cells isolated from aviremic patients who had been under ART treatment for a long period of time. Furthermore, by acting additively with other ARVs, dCA further reduced viral replication by 20% from CD4^+^ T cells isolated from viremic patients. Distinct from any currently available ARVs that prevent new rounds of infection, dCA, a potential groundbreaking compound, inhibits HIV production from integrated proviral DNA, which by its mode of action may drastically reduce the low levels of persistent viremia observed in treated subjects [212]. With a therapeutic index of over 8,000, dCA defines a very promising Tat inhibitor endowed with the ability to decrease residual viremia during ART and should be considered as a potential drug to be included in therapeutic eradication strategies.
